# Supplementary material for: Octopus ‘hypnosis’: inducing tonic immobility for studying local sensorimotor responses and arm-sucker coordination
Source: Biol Open. 2025 Dec 5;14(12):bio062082. doi: 10.1242/bio.062082 (PMC12714133; doi:10.1242/bio.062082)
Supplement: Supplementary information [file biolopen-14-062082-s1.pdf]

Dataset 1.

Available for download at
https://journals.biologists.com/bio/article-lookup/doi/10.1242/bio.062082#supplementary-data

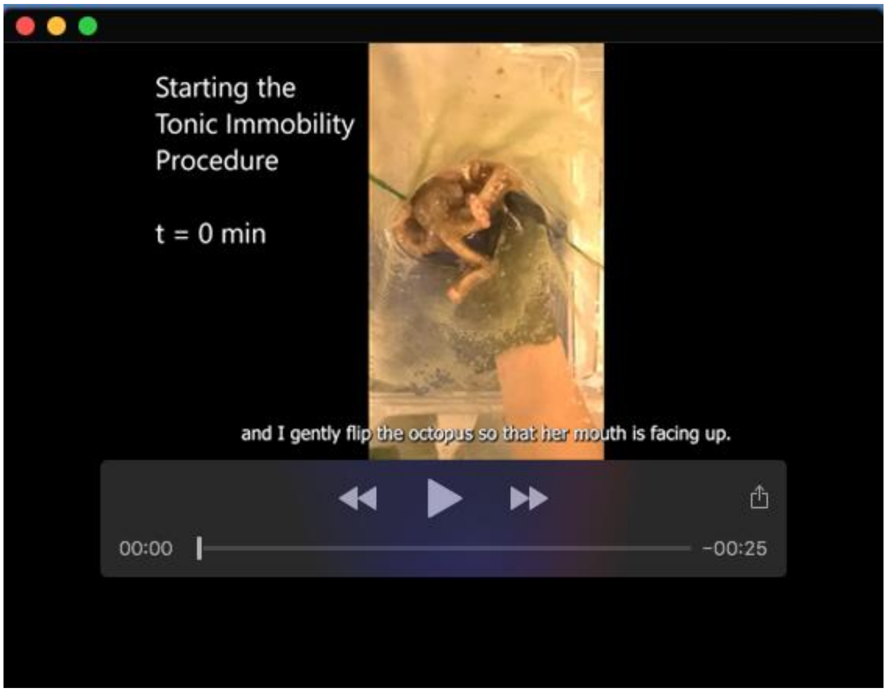

Movie 1. This video shows our modified procedure for octopus “hypnosis”, or tonic immobility, its use in electrode implantation in octopus arms, and lastly the octopus’s quick recovery from tonic immobility.

Biology Open • Supplementary information
